# Sociodemographic factors associated with daily tobacco smoking and binge drinking among Zambians: evidence from the 2017 STEPS survey

**DOI:** 10.1186/s12889-022-12594-2

**Published:** 2022-01-31

**Authors:** Adam Silumbwe, Miguel San Sabastian, Charles Michelo, Joseph Mumba Zulu, Klara Johansson

**Affiliations:** 1 Department of Health Policy and Management, School of Public Health, University of Zambia, PO Box 50110, Lusaka, Zambia; 2 Department of Epidemiology and Global Health, Umeå University, 901 87 Umeå, Sweden; 3 Department of Epidemiology and Biostatistics, School of Public Health, University of Zambia, PO Box 50110, Lusaka, Zambia

**Keywords:** Alcohol, Binge drinking, Socioeconomic, Tobacco, Zambia

## Abstract

**Background:**

The burden of disease attributable to tobacco smoking and harmful alcohol consumption poses a major threat to sustainable development in most low- and middle-income countries. However, evidence on tobacco use and harmful alcohol consumption to inform context-specific interventions addressing these harmful social behaviours is limited in the African context. This study aimed to determine the sociodemographic factors associated with daily tobacco smoking and binge drinking in Zambia.

**Methods:**

The study stems from nationwide population-based representative survey data collected using the World Health Organization’s STEPwise approach for non-communicable disease risk factor surveillance in 2017 among 18–69-year-old Zambians. The main outcomes were daily tobacco smoking and binge drinking, and the demographic and socioeconomic variables included sex, marital status, age, residence, level of education and occupation. Prevalence ratios (PR) were calculated using log-binomial regression analysis.

**Results:**

Overall, 4302 individuals (weighted percentage 49.0% men and 51.0% women) participated in the survey. The prevalence of daily tobacco smoking was 9.0%, and 11.6% of participants engaged in binge drinking, both of which were higher among men than women (17.1% vs. 1.3% and 18.6% vs. 5.3%, respectively). The adjusted prevalence of daily tobacco smoking was 14.3 (95% CI: 9.74-21.01) times higher in men than women, and 1.44 (95% CI 1.03-1.99) times higher in the > 45-year-old group compared to the 18–29-year-old group. Significant positive associations with daily tobacco smoking were found among those with no education 2.70 (95% CI 1.79- 4.07) or primary education 1.86 (95% CI 1.22-2.83) compared to those with senior secondary or tertiary education. The adjusted prevalence of daily tobacco smoking was 0.37 times lower (95% CI 0.16-0.86) among students and homemakers compared to employed participants. The adjusted prevalence of binge drinking was 3.67 times higher (95% CI 2.83-4.76) in men than in women. Significantly lower adjusted prevalences of binge drinking were found in rural residents 0.59 (95% CI: 0.46-0.77) compared to urban residents and in students/homemakers 0.58 (95% CI: 0.35-0.94) compared to employed participants.

**Conclusion:**

This study shows huge differences between men and women regarding tobacco smoking and binge drinking in Zambia. A high occurrence of tobacco smoking was observed among men, older members of society and those with lower levels of education, while binge drinking was more common in men and in those living in urban areas. There is a need to reshape and refine preventive and control interventions for tobacco smoking and binge drinking to target the most at-risk groups in the country.

**Supplementary Information:**

The online version contains supplementary material available at 10.1186/s12889-022-12594-2.

## Background

Tobacco smoking and harmful alcohol consumption are major risk factors for non-communicable diseases (NCDs) [1, 2]. Tobacco smoking alone accounts for more than 8 million global deaths annually, with more than 85% of these deaths the result of direct use [3]. Over 80% of the 1.3 billion tobacco users worldwide live in low- and middle-income countries (LMICs) [4]. Further, harmful alcohol consumption contributes to 3 million deaths annually, and it is responsible for 5.1% of the global burden of disease and injury [5]. In 2016, the alcohol-attributable disease burden was highest in LMICs compared to high-income countries [6].

While considerable gains in reducing tobacco and harmful alcohol consumption have been made, progress has been slow in most LMICs, particularly sub-Saharan Africa (SSA) [7]. The majority of SSA countries remain off-track to meet the global target to cut tobacco use by 30% by 2025 [8]. In addition, SSA faces a growing burden of harmful alcohol consumption [9]. According to the World Health Organization (WHO), SSA is characterized by binge drinking or episodic excessive drinking [10, 11]. The prevalence of binge drinking among current drinkers in SSA is one of the highest in the world, at over 60% [5]. This is largely due to weak policy control measures and an increase in consumer purchasing power providing attractive markets to the tobacco and alcohol industries [12, 13].

This pattern is also reflected in Zambia. Some studies have shown that around 20% of men smoke tobacco, which is higher than most countries in the region [14, 15]. However, this figure is expected to increase because tobacco is a major cash crop prioritized by the government, with incentives provided to bolster its production. Taking advantage of these incentives, multinational companies are expected to produce 5 million cigarettes daily for the Zambian market [16]. Efforts to address tobacco smoking in Zambia have included awareness creation, introduction of tobacco levy, banning of tobacco sales to minors and advertising in the media. These efforts have nevertheless faded over the years. While Zambia signed in 2008 the WHO framework convention on tobacco control (FCTC) and passed the statutory instrument No. 39 which banned smoking in public places [17, 18], several studies have however highlighted the lack of enforcement of these legal frameworks [16, 19, 20]. For instance, in contrast with the FCTC recommended 75% tax share, in 2016 Zambia’s tax comprised only 37% of the retail price of cigarettes [21]. In addition, harmful alcohol consumption is becoming a major problem in the country. The WHO report of 2016 stated that the prevalence of alcohol use disorders among Zambians, including alcohol dependence and harmful use, was 9.8% for males and 1.2% for females, with an overall prevalence of 5.5%, which is above the average of 3.7% for the WHO African Region [22]. Although an alcohol policy was passed in 2018 to reduce harmful use of alcohol [23], it faces major implementation challenges. While the policy emphasises several restrictive measures along the marketing process, no legally binding regulations on alcohol production, distribution, advertising, sponsorship nor sales promotion have been established [22].

Any efforts to minimise the impact of tobacco smoking and harmful alcohol consumption, particularly binge drinking, in Zambia will require a better understanding of their sociodemographic distribution [14]. While some studies have previously explored the issue of tobacco smoking and binge drinking in Zambia, they were either not nationally representative or were conducted on selected subpopulations [24, 25].

This study aimed to determine the sociodemographic factors associated with daily tobacco smoking and binge drinking in a nationally representative sample of Zambian men and women.

## Methods

### The STEPS survey

Data for this study originate from the WHO STEPwise approach to non-communicable diseases risk factor surveillance (STEPS survey) conducted in Zambia in 2017 among adults aged 18–69 years old [26]. The first STEPS effort in Zambia was performed on a small pilot sample in Lusaka and Kitwe in 2008 as formative preparation for the nationwide survey that followed in 2017.

#### Sampling and data collection

The Zambia STEPS survey of 2017 comprised population representative data on NCD risk factors collected using a multistage cluster sampling approach [26]. A total of 5791 households from 347 standard enumeration areas (SEA) were identified using the Zambia Population-based HIV Impact Assessment (ZAMPHIA) survey. The sampling process consisted of three stages. Firstly, SEAs were selected from the 10 provinces using a probability proportional to the population size. Secondly, 15 households in rural and 20 households in urban SEAs were then selected systematically using an appropriate sampling interval based on the number of households in that SEA. Lastly, one eligible member of each household was randomly selected to be interviewed. The STEPS questionnaire was translated into all seven common local Zambian languages [26]. A tablet with the WHO e-STEPS software was used to collect and transmit data to a central server [26]. Details of the STEPS methodology have been reported elsewhere [26].

#### Variable description and measurement

Two outcome variables were selected for this study: daily tobacco smoking and binge drinking. For daily tobacco smoking, participants were asked the question, “do you currently smoke any tobacco products (such as cigarettes, shisha, cigars or pipes) daily?” Daily tobacco smoking was dichotomised into those who did or did not report smoking tobacco daily (yes or no). According to the WHO, binge drinking or heavy episodic drinking is defined as consuming more than five alcoholic drinks for men and four for women on three or more days per week [24]. However, for this study, a proxy measure for binge drinking was derived from the question, “how many times did you have six or more standard drinks in a single drinking occasion over the past 30 days?” [27] Binge drinking was dichotomised into those who responded never and those who answered at least once.

The sociodemographic variables included sex, defined as men and women; marital status, divided into single, married/cohabiting and divorced/separated/widowed; age groups, classified as 18–29, 30–44 and > 44 years; and residence, split into rural and urban locations. Socioeconomic variables in the survey included the level of education (number of years spent in school), classified as no education, primary (7 years), junior secondary (9 years), senior secondary (12 years) and tertiary (13 years or more), and occupation, categorised into student/homemaker, unemployed and employed. Given the small size of some categories, senior secondary education was merged with tertiary education, employed with self-employed and students with homemakers. Although self-reported income was asked in the survey, the large number of missing values meant this variable could not be used.

### Statistical analysis

Both descriptive and inferential statistics were used to determine the prevalence of daily tobacco smoking and binge drinking, as well as the associated sociodemographic factors. Sampling weights accounted for non-response, and sampling procedures were applied to all analyses [28]. Descriptive statistics were calculated using frequencies and proportions. Log-binomial regression models were conducted to estimate crude and adjusted prevalence ratios (PR) with 95% confidence intervals (CI). Given their relevance in the crude analysis, two separate analyses stratified by sex (male and female) and residence (rural and urban) were also conducted (Additional file 1). All analyses were carried out using Stata 14.2 (Stata Corp, College Station, Texas, USA).

### Ethical considerations

The STEPS survey protocol received ethical approval from the University of Zambia Biomedical Research Committee (UNZABREC) and the Zambia National Research Authority (ZNHRA). The survey also received authorisation from the Ministry of Health (MoH) and the WHO country office. Informed consent was obtained from all people who participated in the study. The final dataset was compiled and deposited at the MoH headquarters, Lusaka. To access the secondary data for this study, ethical approval was provided by the ZNHRA and the ERES Converge Ethics Review Board (ref. no. 2019-Dec-007). This research was performed in accordance with the Declaration of Helsinki [29].

## Results

### Participation and distribution

Overall, 4302 participants were included, representing a response rate of 77.7%. The weighted sample was equally represented for sex, with 49.0% men and 51.0% women. More than half (58.9%) of the sample were married or cohabiting, and nearly half (46.9%) were 18–29 years old. There was a similar rural and urban representation, nearly a third (28.8%) had no education and half (50.3%) reported that they were employed. More women than men had no education (34.7% vs. 22.5%). The largest occupational category in both men and women was employed (59. 9% vs 41.0%) (Table 1).

**Table 1:** Weighted description of sociodemographic variables and outcomes in the total sample presented by sex

Variables	Total sample n (%)	Men n (%)	Women n (%)
**Total sample**	4302 (100.0)	2109 (49.0)	2193 (51.0)
**Marital status**			
Single	1286 (30.0)	726 (34.5)	559 (25.6)
Married/cohabitating	2527 (58.9)	1274 (60.5)	1253 (57.3)
Divorced/widowed	480 (11.2)	105 (5.0)	374 (17.1)
**Age (years)**			
18–29	2015 (46.9)	1002 (47.5)	1013 (46.2)
30–44	1470 (34.2)	732 (34.7)	739 (33.7)
> 45	816 (19.0)	375 (17.8)	441 (20.1)
**Residence**			
Urban	2104 (48.9)	970 (46.0)	1134 (51.7)
Rural	2198 (51.1)	1138 (54.0)	1060 (48.3)
**Education level**			
Senior secondary/tertiary	1145 (26.6)	658 (31.2)	487 (22.2)
Junior secondary	929 (21.6)	504 (23.9)	425 (19.4)
Primary	989 (23.0)	470 (22.3)	519 (23.7)
No education	1236 (28.8)	475 (22.5)	761 (34.7)
**Occupation**			
Employed	2159 (50. 3)	1262 (59. 9)	897 (41.0)
Unemployed	1415 (32.9)	578 (27. 4)	837 (38.3)
Student/homemaker	721 (16.8)	267 (12.7)	454 (20.8)
**Daily tobacco smoking**^**a**^			
Yes	389 (9.0)	361 (17.1)	28 (1.3)
No	3912 (91.0)	1748 (82.9)	2164 (98.7)
**Binge drinking**^**b**^			
Yes	468 (11.6)	355 (18.6)	113 (5.3)
No	3578 (88.4)	1557 (81.4)	2021 (94.7)

^a^Daily tobacco smoking: self-reported daily tobacco smoking (cigarettes, shisha, cigars or pipes). ^b^Binge drinking; reporting six or more standard drinks in a single drinking occasion over the past 30 days. Missing values have been omitted from the table

### Daily tobacco smoking and binge drinking prevalence

The overall prevalence of daily tobacco smoking was 9.0%, being higher among men than women (17.1% vs. 1.3%). Higher daily tobacco smoking was reported in > 45-year-old (12.9%), non-educated (12.7%) and employed (11.9%) participants, as well as in rural residents (11.8%) (Table 2).

**Table 2:** Prevalence and sociodemographic factors associated with self-reported daily tobacco smoking and binge drinking in Zambia (2017 STEPS survey)

Variable	Daily tobacco smoking^**a**^			Binge drinking^**b**^		
	Prevalence n (%)	Crude Prevalence Ratio (95% CI)	Adjusted Prevalence Ratio (95% CI)	Prevalence n (%)	Crude Prevalence Ratio (95% CI)	Adjusted Prevalence Ratio (95% CI)
**Sex**						
Women	28 (1.3)	1	1	113 (5.3)	1	1
Men	361 (17.1)	13.34 (9.19, 19.36)*	14.30 (9.74, 21.01)*	355 (18.6)	3.51 (2.74, 4.50)*	3.67 (2.83, 4.76)*
**Marital status**						
Single	83 (6.5)	1	1	128 (10.5)	1	1
Married/cohabitating	272 (10.8)	1.66 (1.19, 2.31)*	1.05 (0.71, 1.55)	298 (12.6)	1.20 (0.90, 1.60)	1.17 (0.83, 1.65)
Divorced/widowed	33 (7.0)	1.07 (0.66, 1.75)	1.30 (0.78, 2.15)	41 (9.12)	0.87 (0.58, 1.30)	1.21 (0.76, 1.93)
**Age (years)**						
18–29	139 (6.9)	1	1	196 (10.2)	1	1
30–44	145 (9.9)	1.43 (1.06,1.93)*	1.07 (0.77, 1.48)	182 (13.4)	1.32 (1.01, 1.71)*	1.10 (0.82, 1.50)
> 45	105 (12.9)	1.87 (1.39, 2.53)*	1.44 (1.03, 1.99)*	90 (11.9)	1.17 (0.87, 1.56)	0.99 (0.71, 1.40)
**Residence**						
Urban	131 (6.2)	1	1	267 (13.5)	1	1
Rural	258 (11.8)	1.89 (1.43, 2.48)*	1.13 (0.84, 1.53)	201 (9.7)	0.72 (0.57, 0.90)*	0.59 (0.46, 0.77)*
**Education level**						
Senior secondary/tertiary	63 (5.5)	1	1	149(13.8)	1	1
Junior secondary	71 (7.7)	1.40 (0.89, 2-21)	1.47 (0.92, 2.34)	100(11.5)	0.83 (0.59, 1.17)	1.04 (0.73, 1.47)
Primary	96 (9.7)	1.76 (1.17, 2.65)*	1.86 (1.22, 2.83)*	91 (9.8)	0.71 (0.51, 0.99)*	0.96 (0.68, 1.35)
No education	157 (12.7)	2.31 (1.59, 3.36)*	2.70 (1.79, 4.07)*	128 (11.0)	0.80 (0.60, 1.07)	1.33 (0.95, 1.86)
**Occupation**						
Employed	258 (11.9)	1	1	278 (14.0)	1	1
Unemployed	114 (8.1)	0.68 (0.53, 0.87)*	0.86 (0.67, 1.10)	148 (10.9)	0.77 (0.60, 0.99)*	1.02 (0.78, 1.33)
Student/homemaker	16 (2.2)	0.19 (0.08, 0.43)*	0.37 (0.16, 0.86)*	42 (6.0)	0.43 (0.27, 0.67)*	0.58 (0.35, 0.94)*

^a^Daily tobacco smoking: self-reported daily tobacco smoking (cigarettes, shisha, cigars or pipes). ^b^Binge drinking; reporting six or more standard drinks in a single drinking occasion over the past 30 days. * *P*-value < 0.05. Missing values have been omitted from the table

Binge drinking was reported in 11.6% of the sample participants, again higher in men than women (18.6% vs. 5.3%). A higher prevalence of binge drinking was reported among employed participants (14.0%), those with senior secondary or tertiary education (13.8%), residents of urban areas (13.5%), those aged 30–44 years (13.4%) and those who were married or cohabiting (12.6%) (Table 2).

### Regression analysis

The adjusted prevalence of daily tobacco smoking was statistically significantly higher 14.3 (95% CI: 9.74-21.01) among men than women, among participants aged > 45 years compared to those aged 18–29 years 1.44 (95% CI: 1.03- 1.99), and among those with no education 2.70 (95% CI: 1.79-4.07) or primary education 1.86 (95% CI: 1.22-2.83) compared to those with senior secondary or tertiary education. The adjusted prevalence of daily tobacco smoking was 0.37 times lower (95% CI 0.16-0.86) among students and homemakers compared to employed participants (Table 2). After stratifying by sex, similar factors were positively associated with smoking in men but not in women. Similarly, in rural areas, men, older age and lower education were positively associated with smoking; while being a student or homeworker was negatively associated. However, in urban areas, only men and a low education level were positively associated with smoking (Additional file 1, Tables 1 and 3).

The adjusted prevalence of binge drinking was 3.67 times higher (95% CI: 2.83-4.76) in men compared to women. Significantly lower adjusted prevalences of binge drinking were found in rural residents 0.59 (95% CI: 0.46-0.77) compared to urban residents and in students/homemakers 0.58 (95% CI: 0.35-0.94) compared to employed participants (Table 2). After stratifying by sex, similar factors were positively associated with binge drinking among men; however, among women, the adjusted prevalence of binge drinking was higher among the unemployed than employed participants 1.62 (95% CI: 1.04-2.54). In both rural and urban areas, being male was positively associated with binge drinking, while the adjusted prevalence of binge drinking was 1.59 times higher (95% CI: 1.03-2.45) among the 30–44 years than those aged 18–29 years only in rural areas (Additional file 1, Tables 2 and 4).

## Discussion

This study investigated the sociodemographic factors associated with daily tobacco smoking and binge drinking among Zambians using a nationally representative sample from the 2017 WHO STEPS survey. The overall prevalence of daily tobacco smoking was 9.0%, while 11.6% of participants engaged in binge drinking, both of which were higher among men than women. Older age and having primary or no education were also significant factors related to daily tobacco smoking. Compared to employed participants, students and homemakers had a lower prevalence of daily tobacco smoking. Binge drinking was positively associated with being male and living in an urban area. Again, students and homemakers had a lower prevalence of binge drinking than employed participants.

Although tobacco smoking trends in Zambia have fluctuated over the years, they clearly indicate a major public health threat, particularly among men. The high prevalence of tobacco smoking amongst Zambian men (17.1%) has been reported in other studies, though slightly higher at 20% [14, 15]. This gender disparity was expected due to the differing social norms that promote tobacco use. Tobacco smoking is much more tolerated in men than women, owing to dominant internalised gender stereotypes of masculinity [30]. In similar lines, other studies have reported that Zambian men have some of the highest smoking rates in the southern African region [31, 32].

This study found that older age was positively associated with tobacco smoking. Similar findings were reported in a systematic review of smoking data from 30 SSA countries [33]. A possible explanation may be that in some SSA cultures, tobacco smoking is viewed as a practice reserved for elderly people [30]. However, other studies in countries such as Kenya and Burkina Faso have reported that tobacco smoking is more common in younger people [34, 35]. Our findings might suggest that tobacco smoking in Zambia may have been more popular among younger groups some years back, resulting in higher consumption among elderly individuals now.

A lower education level was significantly associated with daily tobacco smoking, which confirms the already established association reported in other studies [33, 36, 37]. Indeed, studies have pointed out that poor people and those with a lower education status tend to be missed by large public health prevention efforts [38]. Perhaps this could also be the case in the Zambian setting, where public health interventions such as anti-tobacco smoking awareness creation efforts might only be reaching those with some form of literacy. This calls for a reflection on how to ensure anti-tobacco messages are delivered in the most simple and effective way possible across a spectrum of media platforms.

Compared to the employed, students and homemakers had a lower prevalence of daily tobacco smoking. Similar findings have been reported in studies from other LMIC such as Ethiopia, Madagascar and Nepal [39–41]. This group is, of course, comprised mainly of young people and women, but since we controlled for age and sex, this should not be the reason for the low prevalence. A possible explanation is that both students and homemakers may be subjected to a sociocultural environment that disapproves and frowns upon smoking in this category of tobacco users.

The prevalence of binge drinking was slightly lower than the overall WHO estimate among the Zambian population > 15 years old of 13.5%, again higher in men (23%) than in women (4.1%) [22]. This could be because of the different measurements of binge drinking [26, 27]. The higher level of binge drinking among men compared to women could be explained by the fact that culturally, binge drinking is encouraged among men as a representation of power and strength [42]. Similar patterns of drinking have been reported in studies from South Africa, where the overall prevalence of binge drinking was reported to be 18.3%, and again higher in men than women (22.8% vs. 6.4%) [10, 43]. It is not possible to compare our findings with other Zambian-based studies since these have been conducted on subpopulations such as HIV-positive people, those in psychiatric settings and college students [24, 44, 45]. These studies have found a much higher prevalence than in the general population [24, 44, 45]; for instance, 81.4% among people living with HIV [24].

The prevalence of binge drinking was significantly lower among rural residents compared to those from urban areas. This finding has also been reported in studies from neighbouring countries, such as South Africa [46]. According to Letsela et al., in urban areas, alcohol is easily accessible and highly marketed through advertising in the media which influence its consumption [47, 48]. In the Zambian context, the lack of legally binding regulations on alcohol distribution and advertising, may have also contributed to partly explain our findings [22].

Similar to tobacco use, students and homemakers were less likely to binge drink compared to those in employment. Similar findings have been reported in an Ethiopian study which found that the likelihood of heavy episodic drinking was lower among housewives compared to those employed [49]. One possible explanation could be limited control over money to spend on binge drinking among homemakers and students [50]. However, after stratifying by sex, unemployed women had a higher prevalence of binge drinking than employed women, which highlights the vulnerability of this specific group.

### Strengths and limitations of the study

The major strength of this study was the national representativeness of the data, and consequently, the generalisability of our findings. The STEPS survey uses validated and reliable tools and has a methodologically sound design. Since survey weights are constructed with the aim to build a population-representative sample, this should compensate for the potential non-randomness of drop-outs; however, there may still be residual non-randomness that could bias the results. One limitation of this study is that some of the variables, such as binge drinking, were answered retrospectively, which could be affected by recall bias. Another limitation concerns the question used for binge drinking where the perception of a ‘standard’ drink could be different among participants. The small number of women who engaged in smoking or binge drinking limited the analysis of factors associated with these behaviours. Lastly, we are aware that the merging of small variable categories for education, age and employment may have also affected our estimations. Despite these limitations, our findings are valuable for informing tobacco and alcohol control efforts in Zambia.

## Conclusion

We found a high occurrence of tobacco smoking predominantly among men, elderly and those with a lower education level, while binge drinking was more prevalent in men and urban residents. Overall, rural residents smoked more, and urban residents drank more. Our findings emphasize the importance of local, tailored tobacco and alcohol control interventions to ensure relevance and utility in different Zambian at risk populations and communities. These interventions should be grounded in known family- and community-centred models to sustain efforts against the uptake of these risky social behaviours. Since Zambia has already committed to the FCTC, priority must be given to enforcing the FCTC recommendations such as mandatory tobacco health warnings, implementing plain packaging and enforcing tobacco advertising bans. There is also need for a more effective taxation regime to deter from both smoking and alcohol drinking. Further, multilevel interventions such as regulation of production, distribution, sale and consumption of alcohol in urban areas, including screening and referrals for treatment and counselling services for dependent individuals within primary healthcare facilities are required. Future research should focus on monitoring trends in tobacco smoking and harmful alcohol consumption among at-risk groups as well as exploring the bottlenecks stifling implementation of the existing legal and policy frameworks against tobacco and alcohol in Zambia.

## Supplementary Information

**Additional file 1.**

## Data Availability

All data generated or analysed during this study are included in this published article [and its supplementary information files].

## Abbreviations

MoH: Ministry of health
LMICs: Low-and middle-Income countries
UNZABREC: University of Zambia bioethics research ethics committee
ZNHRA: Zambia national health research authority

## References

[1] Bagnardi V (2015). Alcohol consumption and site-specific cancer risk: a comprehensive dose–response meta-analysis. Br J Cancer.

[2] Prabhu A, Obi KO, Rubenstein JH (2014). The synergistic effects of alcohol and tobacco consumption on the risk of esophageal squamous cell carcinoma: a meta-analysis. Am J Gastroenterol.

[3] World Health Organization (2017). WHO report on the global tobacco epidemic: monitoring tobacco use and prevention policies.

[4] World Health Organization (2020). Tobacco Key Facts.

[5] World Health Organization (2018). Global status report on alcohol and health.

[6] Griswold MG (2018). Alcohol use and burden for 195 countries and territories, 1990–2016: a systematic analysis for the global burden of disease study 2016. Lancet.

[7] Sarah Wild. Smoking has risen 50% in Africa over 35 years even as it drops in high-income regions March 14, 2018. [cited 6th April 2021] Available from: https://qz.com/africa/1228845/africas-smoking-is-up-50-even-as-it-drops-in-wealthy-continents/.

[8] Méndez D, Alshanqeety O, Warner KE (2013). The potential impact of smoking control policies on future global smoking trends. Tob Control.

[9] Ferreira-Borges C (2016). The impact of alcohol consumption on African people in 2012: an analysis of burden of disease. Tropical Med Int Health.

[10] Vellios N, Van Walbeek C (2018). Self-reported alcohol use and binge drinking in South Africa: evidence from the National Income Dynamics Study, 2014-2015. S Afr Med J.

[11] Owolabi EO (2018). Adult binge drinking: rate, frequency and intensity in Buffalo City metropolitan municipality, South Africa. South Afr Fam Pract.

[12] Gilmore AB (2015). Exposing and addressing tobacco industry conduct in low-income and middle-income countries. Lancet.

[13] Jernigan DH, Babor TF (2015). The concentration of the global alcohol industry and its penetration in the a frican region. Addiction.

[14] Yaya S, Bishwajit G (2019). Alcohol and tobacco use among men in Zambia and Zimbabwe. J Lifestyle Med.

[15] Nalule FM, Michelo CC, Olowski P (2020). Tobacco smoking prevalent in Zambian males: observations from the Zambia demographic health survey 2013-2014. Med J Zambia.

[16] Labonté R (2019). Consequences of policy incoherence: how Zambia’s post-FCTC investment policy stimulated tobacco production. J Public Health Policy.

[17] World Health Organization (2004). WHO framework convention on tobacco control.

[18] Zambia Ministry of Local Government (2008). Statutory instrument No 39. Prohibition of smoking in public places.

[19] Labonté R (2018). The institutional context of tobacco production in Zambia. Glob Health.

[20] Lencucha R (2016). Investment incentives and the implementation of the framework convention on tobacco control: evidence from Zambia. Tob Control.

[21] Rowell A, Chamberlain P (2021). Tobacco tactics. Zambia- Country Profile.

[22] World Health Organization (2016). Zambia Country Profile. Alcohol Consumption: Levels and Patterns.

[23] Zambia Ministry of Health (2018). Alcohol Policy and Implementation Plan.

[24] Nouaman MN (2018). High prevalence of binge drinking among people living with HIV in four African countries. J Int AIDS Soc.

[25] Likashi DV, Paul R, Jason L (2019). The proportion of binge drinking among female social drinkers of Kalingalinga in Lusaka, Zambia: a pilot study. Glob Psychiatry.

[26] World Health Organization (2005). The WHO STEPwise approach to chronic disease risk factor surveillance.

[27] Nordqvist C, Johansson K, Bendtsen P (2004). Routine screening for risky alcohol consumption at an emergency department using the AUDIT-C questionnaire. Drug Alcohol Depend.

[28] Heeringa SG, West BT, Berglund PA (2017). Applied survey data analysis.

[29] Association, G.A.o.t.W.M (2014). World medical association declaration of Helsinki: ethical principles for medical research involving human subjects. J Am Coll Dent.

[30] Egbe CO (2014). An exploratory study of the socio-cultural risk influences for cigarette smoking among southern Nigerian youth. BMC Public Health.

[31] Brathwaite R (2015). A systematic review of tobacco smoking prevalence and description of tobacco control strategies in sub-Saharan African countries; 2007 to 2014. PLoS One.

[32] American Cancer Society (2016). Tobacco Atlas.

[33] Sreeramareddy CT, Pradhan PM, Sin S (2014). Prevalence, distribution, and social determinants of tobacco use in 30 sub-Saharan African countries. BMC Med.

[34] Ngaruiya C (2018). Tobacco use and its determinants in the 2015 Kenya WHO STEPS survey. BMC Public Health.

[35] Bonnechère B (2019). Tobacco use and associated risk factors in Burkina Faso: results from a population-based cross-sectional survey. BMC Public Health.

[36] Gilman SE (2008). Educational attainment and cigarette smoking: a causal association?. Int J Epidemiol.

[37] Sreeramareddy CT, Pradhan PMS (2015). Prevalence and social determinants of smoking in 15 countries from North Africa, central and Western Asia, Latin America and Caribbean: secondary data analyses of demographic and health surveys. PLoS One.

[38] Elsey H (2019). Rethinking health systems in the context of urbanisation: challenges from four rapidly urbanising low-income and middle-income countries. BMJ Glob Health.

[39] Lakew Y, Haile D (2015). Tobacco use and associated factors among adults in Ethiopia: further analysis of the 2011 Ethiopian demographic and health survey. BMC Public Health.

[40] Khanal V, Adhikari M, Karki S (2013). Social determinants of tobacco consumption among Nepalese men: findings from Nepal demographic and health survey 2011. Harm Reduct J.

[41] Mamudu HM (2013). The odd man out in sub-Saharan Africa: understanding the tobacco use prevalence in Madagascar. BMC Public Health.

[42] Setlalentoa BMP (2009). The socio–economic effects of binge drinking on support networks in the North–West Province: a social perspective.

[43] World Health Organization (2018). South Africa Alcohol Consumption: Levels and Patterns.

[44] Paul R, Ncheka J, Hammerstein N (2017). Increasing problem of alcohol abuse among the Zambian population in the psychiatric setting.

[45] Chersich M, Rees H (2010). Causal links between binge drinking patterns, unsafe sex and HIV in South Africa: its time to intervene. Int J STD AIDS.

[46] Peltzer K, Davids A, Njuho P. Alcohol use and problem drinking in South Africa: findings from a national population-based survey. Afr J Psychiatry. 2011;14(1):30–7. 10.4314/ajpsy.v14i1.65466 21509408

[47] Letsela L (2019). Alcohol availability, marketing, and sexual health risk amongst urban and rural youth in South Africa. AIDS Behav.

[48] Weitzman ER, Nelson TF, Wechsler H (2003). Taking up binge drinking in college: the influences of person, social group, and environment. J Adolesc Health.

[49] Gutema BT (2020). Prevalence of heavy episodic drinking and associated factors among adults residing in Arba Minch health and demographic surveillance site: a cross sectional study. BMC Public Health.

[50] Ajayi AI, Owolabi EO, Olajire OO (2019). Alcohol use among Nigerian university students: prevalence, correlates and frequency of use. BMC Public Health.

