# Supplementary material for: Sociodemographic factors associated with daily tobacco smoking and binge drinking among Zambians: evidence from the 2017 STEPS survey
Source: BMC Public Health. 2022 Jan 31;22:205. doi: 10.1186/s12889-022-12594-2 (PMC8805266; doi:10.1186/s12889-022-12594-2)
Supplement: Supplementary file 1 — Additional file 1. [file 12889_2022_12594_MOESM1_ESM.docx]

**Additional file 1**

**Table 1:** Prevalence and sociodemographic factors associated with self-reported daily tobacco smoking among men and women in Zambia (2017 STEPS Survey).

| **Variable** | **Tobacco smoking men** | | | **Tobacco smoking women** | | |
| --- | --- | --- | --- | --- | --- | --- |
|  | Prevalence  n (%) | Crude PR  (95% CI) | Adjusted PR (95% CI) | Prevalence  n (%) | Crude PR (CI)  (95% CI) | Adjusted PR (95% CI) |
| **Marital status** |  |  |  |  |  |  |
| Single | 62 (11.2) | 1 | 1 | 2 (0.4.) | 1 | 1 |
| Married/cohabitating | 193 (19.7) | 1.76 (1.26,2.46)* | 1.02 (0.69,1.53) | 25 (1.7) | 4.57 (1.67,12.53)* | 2.48 (0.52,11.84) |
| Divorced/widowed | 21 (26.5) | 2.36 (1.42,3.93)* | 1.37 (0.81,2.32) | 7 (1.5) | 4.05 (1.28,12.77)* | 1.40 (0.26,7.43) |
| **Age range** |  |  |  |  |  |  |
| 18-29 | 101 (13.2) | 1 | 1 | 8 (0.7) | 1 | 1 |
| 30-44 | 104 (18.6) | 1.41 (1.04,1.91)* | 1.06 (0.76,1.48) | 11 (1.2) | 1.81 (0.62,5.32) | 1.28 (0.39,4.24) |
| >45 | 71 (24.7) | 1.87 (1.37,2.54)* | 1.38 (0.98,1.94) | 16 (2.9) | 4.47 (1.67,11.98)* | 2.83 (0.93,8.56) |
| **Residence** |  |  |  |  |  |  |
| Urban | 92 (12.4) | 1 | 1 | 13 (0.9) | 1 | 1 |
| Rural | 184 (21.1) | 1.70 (1.28,2.27)* | 1.15 (0.83,1.57) | 22 (1.7) | 1.79 (0.84,3.82) | 0.90 (0.42,1.92) |
| **Education level** |  |  |  |  |  |  |
| Higher secondary/tertiary | 45 (9.02) | 1 | 1 | 4 (0.8) | 1 | 1 |
| Lower secondary | 54 (14.1) | 1.56 (0.98,2.47) | 1.54 (0.96,2.49) | 1 (0.2) | 0.20 (0.02,2.13) | 0.19 (0.02,2.10) |
| Primary | 70 (19.3) | 2.14 (1.41,3.25)* | 1.91 (1.23,2.95)* | 6 (1.0) | 1.30 (0.28,5.92) | 0.96 (0.20,4.64) |
| No education | 106 (29.2) | 3.24 (2.21,4.74)* | 2.69 (1.76,4.13)* | 23 (2.5) | 3.32 (0.82,13.44) | 1.97 (0.44,8.95) |
| **Occupation** |  |  |  |  |  |  |
| Employed | 190 (19.7) | 1 | 1 | 11 (1.0) | 1 | 1 |
| Unemployed | 74 (16.8) | 0.85 (0.65,1.12) | 0.80 (0.62,1.05) | 21 (2.1) | 1.99(0.87,4.53) | 2.10 (0.96,4.61) |
| Student/homemaker | 11 (5.4) | 0.28 (0.11,0.68)* | 0.37 (0.15,0.92)* | 2 (0.3) | 0.31(0.06,1.61) | 0.44 (0.08,2.44) |

Daily tobacco smoking: self-reported daily tobacco smoking (cigarettes, shisha, cigars or pipes). Missing values have been omitted from the table. * P-value <0.05. Numbers within categories might not always add up to the same in tables with different covariates. This is due to the process of weighting (which is done to compensate for survey design and non-response). When including new covariates, the weighted n may change.

**Table 2:** Prevalence and sociodemographic factors associated with self-reported binge drinking among men and women in Zambia (2017 STEPS Survey).

| **Variable** | **Binge drinking men** | | | **Binge drinking women** | | |
| --- | --- | --- | --- | --- | --- | --- |
|  | Prevalence  n (%) | Crude PR  (95% CI) | Adjusted PR (95% CI) | Prevalence  n (%) | Crude PR (CI)  (95% CI) | Adjusted PR (95% CI) |
| **Marital status** |  |  |  |  |  |  |
| Single | 76 (15.1) | 1 | 1 | 32 (4.8) | 1 | 1 |
| Married/cohabitating | 174 (20.0) | 1.33 (0.95,1.84) | 1.05 (0.72,1.55) | 83 (5.6) | 1.16 (0.68,1.96) | 1.48 (0.73,3.01) |
| Divorced/widowed | 18 (26.9) | 1.78 (1.07,2.95)* | 1.36 (0.78,2.38) | 22 (4.9) | 1.02 (0.54,1.90) | 1.16 (0.53,2.52) |
| **Age range** |  |  |  |  |  |  |
| 18,29 | 113 (16.0) | 1 | 1 | 57 (4.7) | 1 | 1 |
| 30,44 | 104 (21.4) | 1.34 (0.99,1.81) | 1.08 (0.77,1.51) | 54 (6.2) | 1.32 (0.82,2.13) | 1.43 (0.79,2.58) |
| >45 | 52 (20.4) | 1.27 (0.91,1.78) | 1.02 (0.70,1.48) | 27 (5.1) | 1.09 (0.63,1.89) | 1.19 (0.61,2.31)) |
| **Residence** |  |  |  |  |  |  |
| Urban | 137 (20.7) | 1 | 1 | 104 (7.8) | 1 | 1 |
| Rural | 131 (16.8) | 0.81 (0.62,1.06) | 0.70 (0.51,0.94)* | 34 (2.7) | 0.35 (0.22,0.54)* | 0.31 (0.18,0.51)* |
| **Education level** |  |  |  |  |  |  |
| Higher secondary/tertiary | 85 (18.7) | 1 | 1 | 44 (7.5) | 1 | 1 |
| Lower secondary | 60 (17.3) | 0.93 (0.63,1.37) | 1.09 (0.73,1.63) | 25 (5.0) | 0.66 (0.34,1.28) | 0.79 (0.39,1.59) |
| Primary | 50 (15.9) | 0.85 (0.58,1.25) | 0.96 (0.64,1.43) | 29 (4.8) | 0.63 (0.35,1.12) | 0.79 (0.41,1.53) |
| No education | 72 (22.5) | 1.21 (0.86,1.69) | 1.44 (0.99,2.09) | 40 (4.4) | 0.58 (0.34,1.00) | 0.87 (0.44,1.71) |
| **Occupation** |  |  |  |  |  |  |
| Employed | 179 (21.2) | 1 | 1 | 49 (4.7) | 1 | 1 |
| Unemployed | 73 (18.2) | 0.86 (0.63,1.16) | 0.89 (0.65,1.22) | 62 (6.2) | 1.32 (0.84,2.08) | 1.62 (1.04,2.54)* |
| Student/homemaker | 16 (8.0) | 0.38 (0.18,0.77)* | 0.41 (0.19,0.87)* | 26 (4.9) | 1.04 (0.58,1.87) | 1.07 (0.56,2.05) |

Binge drinking; reporting six or more standard drinks in a single drinking occasion over the past 30 days. Missing values have been omitted from the table. * P-value <0.05. Numbers within categories might not always add up to the same in tables with different covariates. This is due to the process of weighting (which is done to compensate for survey design and non-response). When including new covariates, the weighted n may change.

**Table 3**: Prevalence and sociodemographic factors associated with self-reported daily tobacco smoking in Zambia by residence (2017 STEPS Survey).

|  | **Tobacco smoking rural** | | | **Tobacco smoking urban** | | |
| --- | --- | --- | --- | --- | --- | --- |
| **Variable** | Prevalence  n (%) | Crude PR  (95% CI) | Adjusted PR (95% CI) | Prevalence  n (%) | Crude PR (CI)  (95% CI) | Adjusted PR (95% CI) |
| **Sex** |  |  |  |  |  |  |
| Women | 21 (1.7) | 1 | 1 | 8 (0.9) | 1 | 1 |
| Men | 291 (21.1) | 12.71 (8.24,19.60)* | 13.84 (8.92,21.49)* | 94 (12.4) | 13.36 (6.74,26.48)* | 16.00 (7.72,33.18)* |
| **Marital status** |  |  |  |  |  |  |
| Single | 52 (9.7) | 1 | 1 | 31 (4.8) | 1 | 1 |
| Married/cohabitating | 232 (12.7) | 1.32 (0.87,1.98) | 0.86 (0.54,1.37) | 63 (7.9) | 1.65 (0.95,2.87) | 1.66 (0.86,3.21) |
| Divorced/widowed | 28 (9.6) | 0.99 (0.55,1.80) | 1.11 (0.59,2.12) | 8 (4.32) | 0.90 (0.40,2.05) | 1.56 (0.65,3.75) |
| **Age range** |  |  |  |  |  |  |
| 18-29 | 101 (8.8) | 1 | 1 | 43 (5.2) | 1 | 1 |
| 30-44 | 115 (12.0) | 1.36 (0.96,1.91) | 1.22 (0.84,1.77) | 39 (7.4) | 1.42 (0.82,2.47) | 0.75 (0.43,1.33) |
| >45 | 96 (17.3) | 1.96 (1.40,2.75)* | 1.82 (1.26,2.64)* | 20 (7.2) | 1.40 (0.76,2.56) | 0.70 (0.37,1.35) |
| **Education level** |  |  |  |  |  |  |
| Higher secondary/tertiary | 22 (8.0) | 1 | 1 | 35 (4.9) | 1 | 1 |
| Lower secondary | 42 (8.5) | 1.06 (0.54,2.07) | 1.17 (0.61,2.23) | 29 (7.1) | 1.44 (0.75,2.78)) | 1.69 (0.90,3.17) |
| Primary | 86 (12.2) | 1.53 (0.81,2.88) | 1.67 (0.91,3.07) | 19 (6.1) | 1.24 (0.63,2.43) | 1.67 (0.87,3.22) |
| No education | 162 (13.7) | 1.72 (0.94,3.16) | 2.21 (1.23,3.97)* | 18 (8.9) | 1.82 (0.98,3.37) | 3.86 (2.18,6.83)* |
| **Occupation** |  |  |  |  |  |  |
| Employed | 200 (15.0) | 1 | 1 | 72 (8.8) | 1 | 1 |
| Unemployed | 105 (10.5) | 0.70 (0.53,0.92)* | 0.90 (0.69,1.17) | 21 (4.7) | 0.53 (0.31,0.93)* | 0.77 (0.42,1.42) |
| Student/homemaker | 5 (1.7) | 0.12 (0.03,0.39)* | 0.21 (0.06,0.71)* | 9 (2.5) | 0.28 (0.10,0.83)* | 0.53 (0.17,1.62) |

Daily tobacco smoking: self-reported daily tobacco smoking (cigarettes, shisha, cigars or pipes). Missing values have been omitted from the table. * P-value <0.05. Numbers within categories might not always add up to the same in tables with different covariates. This is due to the process of weighting (which is done to compensate for survey design and non-response). When including new covariates, the weighted n may change.

**Table 4**: Prevalence and sociodemographic factors associated with self-reported binge drinking in Zambia, by residence (2017 STEPS Survey).

|  | **Binge drinking rural** | | | **Binge drinking urban** | | |
| --- | --- | --- | --- | --- | --- | --- |
| **Variable** | Prevalence  n (%) | Crude PR  (95% CI) | Adjusted PR (95% CI) | Prevalence  n (%) | Crude PR (CI)  (95% CI) | Adjusted PR (95% CI) |
| **Sex** |  |  |  |  |  |  |
| Women | 34 (2.7) | 1 | 1 | 67 (7.8) | 1 | 1 |
| Men | 209 (16.8) | 6.27 (4.21,9.34)* | 6.99 (4.61,10.60)* | 143 (20.7) | 2.66 (1.93,3.68)* | 2.69 (1.91,3.79)* |
| **Marital status** |  |  |  |  |  |  |
| Single | 38 (7.4) | 1 | 1 | 75 (12.2) | 1 | 1 |
| Married/cohabitating | 179 (10.5) | 1.43 (0.86,2.36) | 1.10 (0.60,2.01) | 118 (15.7) | 1.29 (0.90,1.85) | 1.26 (0.83,1.90) |
| Divorced/widowed | 26 (9.3) | 1.27 (0.67,2.40) | 1.76 (0.84,3.67) | 15 (8.9) | 0.73 (0.43,1.25) | 0.92 (0.51,1.69) |
| **Age range** |  |  |  |  |  |  |
| 18-29 | 80 (7.3)) | 1 | 1 | 102 (12.7) | 1 | 1 |
| 30-44 | 111 (12.4) | 1.70 (1.16,2.50)* | 1.59 (1.03,2.45)* | 70 (14.6) | 1.14 (0.80,1.64) | 0.85 (0.55,1.29) |
| >45 | 51 (10.1) | 1.39 (0.92,2.08) | 1.26 (0.79,2.00) | 37 (14.0) | 1.10 (0.72,1.67) | 0.84 (0.52,1.37) |
| **Education level** |  |  |  |  |  |  |
| Higher secondary/tertiary | 30 (11.5) | 1 | 1 | 97 (14.4) | 1 | 1 |
| Lower secondary | 45 (9.5) | 0.83 (0.47,1.49) | 0.94 (0.53,1.66) | 50 (13.1) | 0.91 (0.59,1.40) | 1.03 (0.67,1.58) |
| Primary | 55 (8.4) | 0.73 (0.42,1.27) | 0.78 (0.45,1.34) | 35 (12.0) | 0.83 (0.54,1.28) | 1.02 (0.66,1.57) |
| No education | 113 (10.2) | 0.89 (0.54,1.47) | 1.12 (0.68,1.85) | 27 (13.8) | 0.96 (0.61,1.53) | 1.45 (0.91,2.30) |
| **Occupation** |  |  |  |  |  |  |
| Employed | 147 (12.0) | 1 | 1 | 122 (16.2) | 1 | 1 |
| Unemployed | 87 (9.1) | 0.76 (0.55,1.04) | 0.96 (0.69,1.35) | 59 (13.5) | 0.83 (0.56,1.22) | 1.00 (0.67,1.48) |
| Student/homemaker | 8 (2.7) | 0.23 (0.09,0.60)* | 0.37 (0.13,1.06) | 28 (7.8) | 0.48 (0.29,0.81)* | 0.59 (0.34,1.04) |

Binge drinking; reporting six or more standard drinks in a single drinking occasion over the past 30 days. Missing values have been omitted from the table. * P-value <0.05. Numbers within categories might not always add up to the same in tables with different covariates. This is due to the process of weighting (which is done to compensate for survey design and non-response). When including new covariates, the weighted n may change.
